# Stabilising the Lattice Oxygen Oxidation Mechanism Through Iridium Single Atoms in Chromium‐Doped Cobalt Iron Layered Double Hydroxides

**DOI:** 10.1002/advs.77198

**Published:** 2026-08-14

**Authors:** Parisa Eskandari, Shujie Zhou, Jodie Yuwono, Yufei Zhao, Hanyu Xu, Zhihong Tian, Jingwen Ba, Ming Zhang, Bernt Johannessen, Thanh Son Bui, Richard F. Webster, Jing Chu, Xunyu Lu, Rose Amal

**Affiliations:** ^1^ School of Chemical Engineering University of New South Wales UNSW Sydney Sydney New South Wales Australia; ^2^ School of Chemical Engineering Adelaide University Adelaide South Australia Australia; ^3^ Centre For Clean Energy Technology University of Technology Sydney Broadway New South Wales Australia; ^4^ Engineering Research Center For Nanomaterials Henan University Kaifeng People's Republic of China; ^5^ Australian Synchrotron‐ANSTO Clayton, Victory Australia; ^6^ Electron Microscope Unit Mark Wainwright Analytical Centre UNSW Sydney Sydney New South Wales Australia; ^7^ School of Materials Science and Engineering UNSW Sydney Sydney New South Wales Australia

**Keywords:** cobalt, dissolution, doping, hydroxide, iridium, layered double hydroxides, overpotential, oxygen evolution, oxygen, transition metal

## Abstract

Transition metal hydroxides are among the most promising alkaline oxygen evolution reaction (OER) catalysts for anion exchange membrane water electrolysers (AEMWEs), owing to their high intrinsic activity originated from the kinetically favourable lattice oxygen oxidation mechanism (LOM). However, lattice oxygen participation often accelerates catalyst degradation through active‐site dissolution, posing a major challenge to the long‐term stability. Herein, we report a synergistic catalyst design that simultaneously promotes efficient LOM and improves durability through incorporating Ir single atoms (Ir_SAs_) and Cr doping into CoFe layered double hydroxide (LDH). The resulting Ir_SAs_/CoFeCr LDH exhibits low overpotential of 252 mV at 10 mA cm^−2^ and maintains stable operation for over 100 h at 500 mA cm^−2^. Combined experimental and theoretical analyses reveal that Ir and Co serve as primary OER‐active sites, while Cr enhances the structural stability by enriching the electron density of neighbouring metal sites. This electronic modulation suppresses over‐oxidation and facilitates lattice oxygen regeneration, while Ir incorporation strengthens the metal‐oxygen covalency, enabling reversible lattice‐oxygen participation. The AEMWE exhibits a low cell voltage of 1.61 V at 1 A cm^−2^ and durability over 120 h with a negligible decay rate of 0.04 mV h^−1^, highlighting the practical viability of the catalyst design for alkaline water electrolysis.

## Graphical abstract

Iridium single atoms and Cr doping synergistically activate and stabilise the lattice oxygen oxidation mechanism in CoFe layered double hydroxides. Electronic modulation enhances metal–oxygen covalency, suppresses over‐oxidation, and enables durable oxygen evolution, delivering high‐performance anion exchange membrane water electrolysis with industrially relevant activity and stability.

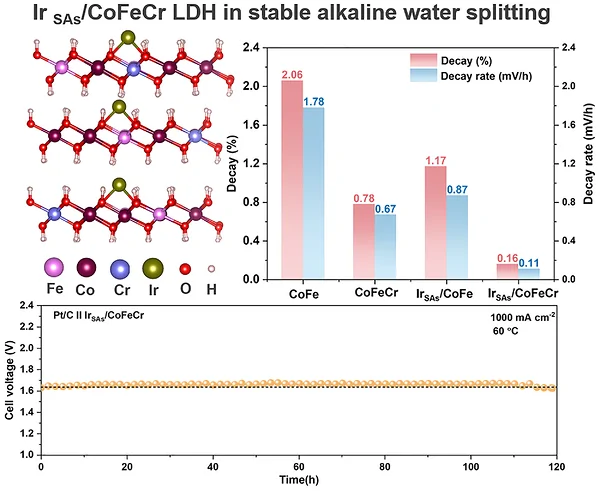

## 1 Introduction

Electrocatalytic water splitting is a promising strategy for sustainable hydrogen production, attracting widespread research interest. Anion exchange membrane water electrolysers (AEMWEs) have attracted significant attention due to their low operational costs and excellent durability [1, 2, 3]. However, the anodic oxygen evolution reaction (OER), as a half‐reaction of water splitting involves a complex four‐electron transfer, leading to sluggish kinetics and a high energy barrier [4, 5]. A fundamental understanding of the OER mechanism is crucial for enhancing kinetics and developing efficient, durable electrocatalysts. In the conventional adsorbate evolution mechanism (AEM), the adsorption energies of OER intermediates (^*^OH and ^*^OOH) follow a linear relationship (ΔG_OOH_ = ΔG_OH_ + 3.2 ± 0.2 eV), imposing a theoretical overpotential limit of ∼370 mV [6, 7, 8]. The recently introduced lattice oxygen oxidation mechanism (LOM) involves lattice oxygen activation and redox during water oxidation, effectively overcoming the linear scaling limitations of AEM and lowering the energy barrier [9]. Consequently, the development of robust and durable LOM‐based OER electrocatalysts has become a critical focus in water‐splitting research.

Transition‐metal oxides and (oxy)hydroxides including layered double hydroxide (LDH) catalysts, have been widely studied to trigger the LOM in OER [10, 11, 12]. However, the lattice oxygen participated in oxygen generation can cause metal leaching, phase transitions, and structural instability, posing a major challenge in achieving both high catalytic activity and long‐term stability, particularly under high‐current‐density conditions [13, 14]. Recent studies have demonstrated that doping with high‐valence metals is an effective strategy to enhance the OER activity and stability of CoFe or NiFe LDHs by tuning the valence state and d‐band centre of active metals and facilitating the LOM pathway. For example, Ce incorporation into NiFe LDH induces charge redistribution through Ce 4f‐O 2p interactions, enabling controlled and localised lattice‐oxygen redox while stabilising Fe active sites against over‐oxidation and leaching [15]. Moreover, the study of Yu et al. on the FeNi_2_S_4_@Cr‐NiFe LDH catalyst shows that Cr doping enriches Ni/Fe with electrons, strengthens the built‐in electric field, and upshifts the d‐band centre, thereby accelerating charge transfer and optimising intermediate adsorption [16]. Besides, Cr doping has also been proved to enhance OER activity in CoFe LDHs by modulating the electronic structure. The formation of Cr^3+^ improves charge transfer via electron donation, while Cr^6+^ enhances electron withdrawal, stabilising high‐valence Co active sites and facilitating more energetically favourable H_2_O adsorption [17, 18]. Despite the rapid progresses over the past decades, the OER performance of CoFeCr LDH is still struggling to meet the requirements for large‐scale AEMWE applications, which are expected to achieve more than 5000 h durability and current densities above 2 A cm^−2^ at cell voltages below 2 V for high energy efficiency and low hydrogen production cost [19]. In practice, the relatively high OER overpotentials and limited durability of transition metal‐based catalysts including CoFeCr LDH result in elevated cell voltages, increased electricity consumption, and accelerated stack degradation, thereby increasing both operating and capital costs. Therefore, strategies that simultaneously reduce OER overpotential and enhance catalyst stability are essential to extend stack lifetime, lower energy consumption, and meet the cost targets (<100 USD kW^−1^ for the stack and <200 USD kW^−1^ for the system) required for MW‐scale AEMWE deployment [19]. In this context, incorporating noble‐metal single atoms (SAs) has emerged as a promising approach because it maximises atomic utilisation of noble metals, enabling significant performance enhancement with minimal noble‐metal loading [20, 21]. However, understanding the collaborative yet distinct roles of SAs and the transition metal elements in the LDHs remains a critical and challenging task, especially how they facilitate the LOM pathway and contribute to high catalytic performance and stability applicable to industrial‐level operation.

In this study, we investigate the impact of incorporating Ir SAs and doping Cr into CoFe LDHs on advancing the alkaline OER performance. The incorporation of Ir SAs further activates the Co─O─Cr coordination as the active sites and enhancing metal‐oxygen covalency, while Cr modulates the electronic structure of Co and Fe toward a more electron‐rich state, it suppresses excessive over‐oxidation, thereby improving the structural stability of the catalyst. Furthermore, incorporating Ir and doping Cr facilitate the oxygen vacancy formation and regeneration in the LOM process, contributing to an accelerated reaction kinetics to boost OER performance. As a result, Ir_SAs_/CoFeCr LDH exhibits highly active and durable OER over 120 h at an industrially relevant current density of 1 A cm^−2^ with negligible decay rate in AEMWEs. This work provides a promising strategy through triggering the LOM process while enhancing the stability for the development of efficient electrocatalysts for industrial‐scale water electrolysis.

## 2 Results and Discussion

### 2.1 Catalyst Preparation and Characterisation

The procedure of synthesis and preparation of Ir_SAs_/CoFeCr LDH is shown in Figure S1 (details provided in the Experimental Section). First, CoFe and CoFeCr LDH were prepared via a co‐precipitation process at room temperature. The obtained CoFeCr powder was then added into 0.01 M NaOH contains 0.1 mmol hydrogen hexachloroiridate(IV) hydrate (H_2_IrCl_6_) as Ir precursor. After 12 h of stirring at room temperature, Ir SAs were successfully anchored onto CoFeCr LDH, forming Ir_SAs_/CoFeCr LDH (Figure S1). The structural and morphological characteristics of the catalyst were investigated through several characterisation techniques. The ‐ray diffraction (XRD) was conducted to study the crystal structure of Ir_SAs_/CoFeCr LDH (Figure S2). The diffraction peaks are assigned to the (003), (006), (012), (104), (015), (107), (018), (110), and (113) crystal planes (JCPDS Card no. 50–0235, confirming the presence of the LDHs structure [22]. It is also observed that introducing Cr slightly reduces the crystallinity of CoFeCr LDH, which can be due to lattice distortion, leading to more amorphous structure that is consistent with the literature [23, 24]. No additional diffraction peaks appear, indicating that Cr is integrated within the LDH lattice rather than forming separate phases. Additionally, there is no change in XRD patterns of CoFe LDH and CoFeCr LDH structure after introducing Ir with no detectable peaks corresponding to Ir. This suggests the absence of Ir nanoparticles, or its content is below the detection limit. Inductively coupled plasma (ICP) analysis confirms Ir and Cr contents of 3.06 and 3.19 wt.% in the Ir_SAs_/CoFeCr LDH while the amount of Co and Fe loading is 30.59 and 10.88 wt.% respectively. Besides, the morphology of the Ir_SAs_/CoFeCr LDH studied by transmission electron microscopy (TEM) confirming the formation of nanosheet structure (Figure 1a). Aberration corrected high angle annular dark‐field scanning transmission electron microscopy (HAADF‐STEM) images of Ir_SAs_/CoFeCr LDH show the presence of atom‐sized bright dots exhibiting the presence of Ir single atoms (marked by red circles) dispersed on CoFeCr (Figure 1b). The lattice fringes, with interplanar distances of 0.16 and 0.19 nm, correspond to the (110) and (012) planes of CoFeCr LDH (Figure S3), respectively, confirming its crystalline structure [17]. Moreover, energy‐dispersive x‐ray spectroscopy (EDS) mapping (Figure 1d–h) demonstrates the presence of Co, Fe, Cr, and Ir, revealing a uniform elemental distribution. The absence of nanoparticle formation or aggregation of Ir and the low distribution density of Ir strongly suggest that Ir exists as atomically dispersed SAs. The intensity in the HAADF‐STEM originates from Rutherford scattering and follows a proportional relationship with the square of the atomic number (Z^2^). Therefore, intensity profiles from different regions can be utilised to identify the atomic species in single‐atom catalysts [25]. The intensity profiles of different regions shown in Figure 1c and Figure 1i indicate the presence of atomic species. Considering their atomic numbers, the brighter atoms can be assigned to atomic Ir, while those with lower intensities correspond to Co, Fe or Cr. However, the similarity in atomic numbers among Co, Fe, and Cr leads to nearly identical intensities, making it unlikely to distinguish these elements and accurately determine how Ir is anchored and interacted on the LDH with Co, Fe, or Cr. Therefore, further characterisation analyses and calculations such as X‐ray absorption spectroscopy (XAS) and density functional theory (DFT) are needed to deeply understand the structure of Ir_SAs_/CoFeCr LDH.

**FIGURE 1:**
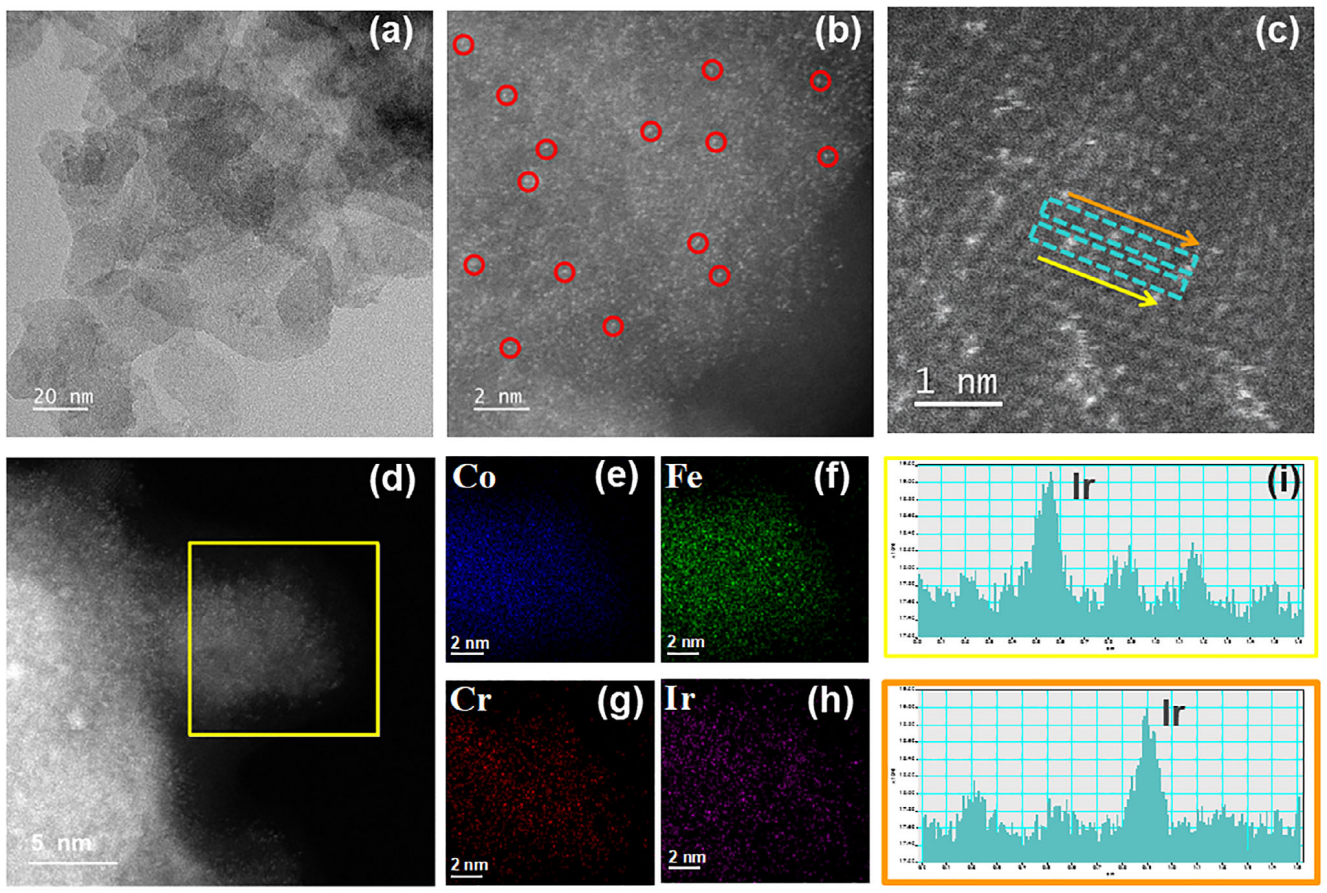
(a) TEM image of Ir_SAs_/CoFeCr LDH, (b) Aberration corrected HAADF‐STEM image (Ir atoms were marked with red circles), (c,i) Intensity profiles of Ir_SAs_/CoFeCr LDH (yellow and orange linear profile scan of different regions of the catalyst).and (d–h) EDS mapping images of Ir_SAs_/CoFeCr LDH.

XAS was employed to probe the local coordination environment and electronic structure of Ir SAs within the CoFeCr LDH matrix. The X‐ray absorption near edge structure (XANES), k^3^‐weighted extended X‐ray absorption fine structure (EXAFS), and associated Fourier transform (FT EXAFS) of the catalysts are shown in Figure 2 and Figure S4. The Ir L_3_‐edge XANES spectra (Figure 2a and Figure S5) show that the absorption edge of Ir_SAs_/CoFeCr LDH lies between those of Ir foil and IrO_2_, indicating a partially oxidised Ir species and the average oxidation state of Ir in the Ir_SAs_/CoFeCr and Ir_SAs_/CoFe catalyst is about 2.50 and 2.73 respectively. Notably, the edge position and white‐line intensity more closely resemble IrO_2_, suggesting a relatively high oxidation state and strong hybridisation with surrounding ligands. We note that XANES edge positions provide qualitative rather than quantitative insight into oxidation state, as they are also influenced by coordination geometry and covalency. At the Co and Fe K‐edges (Figure 2c,d), Cr incorporation induces a shift of the absorption edge to lower energy relative to Ir_SAs_/CoFe, consistent with increased electron density at the Co and Fe centres. This indicates that Cr doping modulates the electronic structure of the LDH host, which in turn influences the electronic interaction with the anchored Ir species.

**FIGURE 2:**
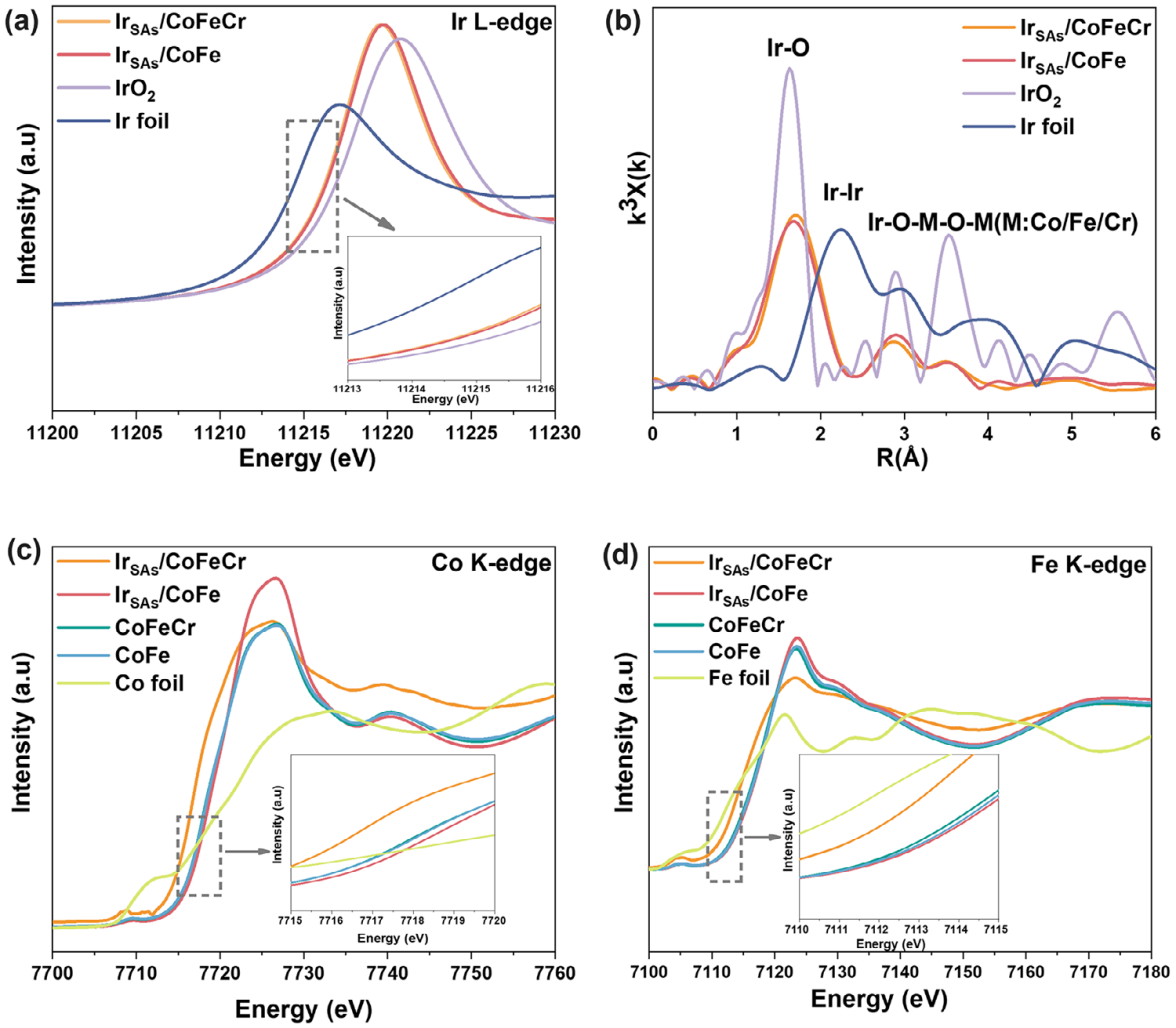
(a) XANES of Ir_SAs_/CoFe and Ir_SAs_/CoFeCr, Ir foil and IrO_2_ at Ir L‐edge, (b) Fourier transform of the k^3^χ(k) EXAFS (without phase correction) of Ir_SAs_/CoFe and Ir_SAs_/CoFeCr, Ir foil and IrO_2_ at Ir L‐edge, (c,d) XANES of Co foil, Fe foil, CoFe, CoFeCr, Ir_SAs_/CoFe and Ir_SAs_/CoFeCr at Co K‐edge and Fe K‐edge.

The Fourier‐transformed EXAFS spectra at the Ir L_3_‐edge (Figure 2b and Figure S4) exhibit a peak at ∼1.6 Å attributed to Ir─O coordination, and a secondary feature at ∼2.7 Å associated with Ir─O─M (M═Co/Fe/Cr) multiple‐scattering contributions [26]. Importantly, no discernible Ir‐Ir scattering is observed near ∼2.50 Å [27], excluding the presence of metallic Ir nanoparticles and supporting atomic dispersion of Ir species, consistent with TEM results. Additionally, X‐ray photoelectron spectroscopy (XPS, Figure S6) reveals that, relative to CoFe, CoFeCr exhibits a negative shift in both Co and Fe binding energies, indicating Cr‐induced electron enrichment of Co and Fe via lattice‐level charge redistribution arising from electron donation by high‐valence Cr. This electron enrichment enhances charge delocalisation within the LDH structure and stabilises the electronic states of Co and Fe, thereby suppressing excessive over‐oxidation and enhancing the regeneration of lattice oxygen under anodic OER conditions [15, 17, 28]. Besides, comparison of the XPS spectra of CoFeCr LDH and Ir_SAs_/CoFeCr LDH reveals a positive shift in the binding energy of both Co and Fe, indicating electron transfer from the CoFeCr LDH to the Ir centres. This electron redistribution implies strong electronic coupling between Ir and the LDH support, leading to a modified electronic structure of the catalyst which is in consistent with EXAFS results. The resulting enhancement in metal‐oxygen (M─O) covalency is expected to facilitate lattice oxygen activation and promote efficient regeneration of lattice oxygen during the OER [29]. Therefore, the electronic modulation induced by Cr doping and Ir single‐atom incorporation can enhance metal‐oxygen covalency and enable efficient charge compensation through lattice oxygen. This electronic regulation can activate lattice‐oxygen redox, promote a‐ LOM‐dominated OER pathway, and simultaneously suppress excessive metal over‐oxidation and improve the regeneration of lattice oxygen, resulting in enhanced OER activity and long‐term structural stability.

### 2.2 Electrocatalytic Performance for OER

The alkaline OER activity of Ir_SAs_/CoFeCr was compared with that of CoFe, CoFeCr and Ir_SAs_/CoFe to evaluate the effect of Ir incorporating and Cr doping. The linear sweep voltammetry (LSV) curves (Figure 3a) measured in 1.0 M KOH solution show that Ir_SAs_/CoFeCr exhibits promising activity with low overpotential of 252 mV required to reach the current density of 10 mA cm^−2^ (η_10_). By contrast, η_10_ for Ir_SAs_/CoFe, CoFeCr, and CoFe, is 273, 280 and 290 mV respectively, implying the positive role of Ir incorporating and Cr doping in enhancing OER activity. Moreover, the Tafel slopes of Ir_SAs_/CoFeCr, Ir_SAs_/CoFe, CoFeCr and CoFe are presented in Figure 3b. The Ir_SAs_/CoFeCr catalyst exhibits a Tafel slope of 45 mV dec^−1^, which is lower than that of Ir_SAs_/CoFe (58 mV dec^−1^), CoFeCr (68 mV dec^−1^) and CoFe (85 mV dec^−1^). The lower Tafel slope suggests enhanced OER kinetics, highlighting the fastest kinetic rate and superior catalytic performance of the Ir_SAs_/CoFeCr among the samples tested. Moreover, electrochemical impedance spectroscopy (EIS) measurements were performed to investigate the interfacial charge transfer resistance of the catalysts. Ir_SAs_/CoFeCr exhibits the smallest semicircle diameter compared to that of Ir_SAs_/CoFe, CoFeCr and CoFe implying its fastest charge transfer at the interface between electrodes and electrolyte (Figure S7) [30]. Additionally, the electrochemical surface area (ECSA) of these catalysts was evaluated in the non‐Faradaic potential region by measuring the double‐layer capacitance (C_dl_) (Figure S8). Ir_SAs/_CoFeCr shows the C_dl_ of 48.08 mF cm^−2^ which is higher than that of Ir_SAs_/CoFe (45.29 mF cm^−2^), CoFeCr (29.48 mF cm^−2^) and CoFe (21.76 mF cm^−2^). The ECSA values were determined based on C_dl_ and the results confirm that Ir_SAs_/CoFeCr leads to an ECSA of over 1200 cm^2^, possessing the highest availability of catalytic active sites. The higher C_dl_ and hence ECSA values of both Ir_SAs_/CoFeCr and Ir_SAs_/CoFe compared to those of CoFeCr and CoFe suggest the vital role of Ir in increasing the exposure of active centres for OER. Moreover, the turnover frequency (TOF) and mass activity of Ir_SAs_/CoFeCr are 1.92 s^−1^ and 3.84 A mg^−1^ respectively, which are twice higher than those of Ir_SAs_/CoFe, confirming the high intrinsic activity of the Ir_SAs_/CoFeCr catalyst. In addition to OER activity, the electrocatalytic stability was evaluated by chronopotentiometry at 500 mA cm^−2^ (Figure 3c), with decay rates and decay percentages identified (Figure 3d). CoFe exhibits poor stability during chronopotentiometry operation at 500 mA cm^−2^, which continues potential increasing remarkably in 25 h with a high decay rate of 1.78 mV h^−1^ and a decay ratio of 2.06% at 25 h. Cr doping markedly enhances durability, reducing the decay rate by ∼2.7 times (to 0.67 mV h^−1^) and the total decay ratio by ∼2.6 times (to 0.78%) for CoFeCr catalyst compared to CoFe, indicating substantially improved stability upon Cr doping. The introduction of Ir SAs further suppresses degradation, with Ir_SAs_/CoFeCr displaying the lowest decay rate (0.11 mV h^−1^) and decay percentage (0.16%) among all samples. This superior stability is maintained upon extended operation, with Ir_SAs_/CoFeCr exhibiting minimal degradation at 50 h (0.18 mV h^−1^, 0.53%), corresponding to ∼5.1‐fold and ∼4.7‐fold lower decay rates and decay percentages, respectively, compared with Ir_SAs_/CoFe (0.91 mV h^−1^, 2.47%). Notably, Ir_SAs_/CoFeCr retains a low decay rate (0.34 mV h^−^^1^) and a limited overall decay (1.93%) over 100 h at high current density, confirming its excellent long‐term stability under industrially relevant conditions. The stability results confirm the vital role of Cr in enhancing the durability of Ir_SAs_/CoFeCr and making the structure of the catalyst more stable. Electrochemical results highlight the excellent OER performance of Ir_SAs_/CoFeCr in alkaline media, confirming the pivotal roles of Ir SAs, Co, and Cr in increasing the availability of active sites and enhancing both activity and stability.

**FIGURE 3:**
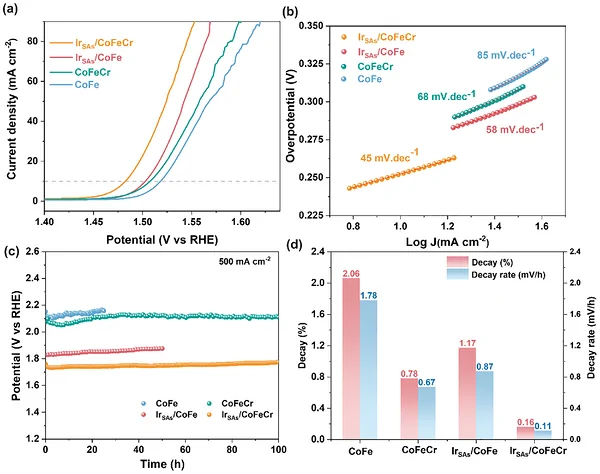
(a) LSV polarisation curves, (b) Tafel slope of CoFe, CoFeCr, Ir_SAs_/CoFe and Ir_SAs_/CoFeCr, (c) Comparison the stability of CoFe, CoFeCr, Ir_SAs_/CoFe and Ir_SAs_/CoFeCr at 500 mA cm^−2^ and (d) Decay ratio (left axis) and decay rate (right axis) of Ir_SAs_/CoFeCr and the controlled samples.

### 2.3 Electrocatalytic Performance for Overall Water Splitting

Inspired by the superior OER performance of Ir_SAs_/CoFeCr in a three‐electrode system, its electrochemical evaluation was further conducted under more demanding conditions using a two‐electrode configuration electrolyser (Pt/C‖Ir_SAs_/CoFeCr), resembling industrial AEMWE. The polarisation curve of Pt/C‖Ir_SAs_/CoFeCr, presented in Figure 4a, demonstrates that for overall water splitting, Pt/C‖Ir_SAs_/CoFeCr achieves a current density of 1 A cm^−2^ at a low cell voltage of only 1.61 V at 60 °C, which is even better than the most of the previously reported noble‐based electrocatalysts (Figure 4b and Table S1) [31, 32, 33, 34, 35, 36, 37, 38, 39, 40, 41, 42, 43, 44, 45, 46, 47, 48, 49, 50, 51]. Pt/C‖Ir_SAs_/CoFeCr also shows the charge transfer resistance of 0.025 Ω (Figure S9 and Table S2). In addition, the Pt/C‖Ir_SAs_/CoFeCr exhibits high performance to perform stably under industrial conditions for more than 120 h at the industrially required current density as high as 1000 mA cm^−2^ with negligible decay rate of 0.04 mV/h (Figure 4c), implying that the Ir_SAs_/CoFeCr catalyst potentially meets the needs of industrial catalytic applications. Moreover, to further confirm the catalyst durability under more demanding conditions approaching practical AEMWE operation, an additional chronopotentiometric stability test was performed at a current density of 2000 mA cm^−2^ for around 24 h. Pt/C‖Ir_SAs_/CoFeCr exhibited a slight increase in cell voltage throughout the test with the decay rate of 3.8 mV/h and decay ratio of around 4.9% (Figure S10). To further investigate the structural stability of the catalyst, electrolyte samples were collected after around 24 h of AEMWE reaction and analysed by ICP‐MS. The results indicate negligible Co leaching (0.02%) and Ir dissolution (1.0%), and Cr (4.11%) relative to the initial catalyst loading, demonstrating the excellent stability of the catalytically active Co and Ir sites during prolonged electrolysis (Table S3). These results demonstrate that the Cr species are effectively retained within the LDH lattice under alkaline operating conditions, with only limited dissolution after prolonged electrolysis. Fe was excluded from the quantitative analysis because its concentration is significantly influenced by the background Fe content of the alkaline electrolyte. Moreover, the XANES spectra of Ir/CoFeCr at the Ir L‐edge before and after OER were conducted to investigate changes in the electronic structure of Ir. The XANES spectra of Ir/CoFeCr at the Ir L‐edge before and after OER were conducted to investigate changes in the electronic structure of Ir. The XANES post‐OER Ir_SAs_/CoFeCr spectrum (Figure S11) shows a positive shift from 11216.2 to 11216.6 eV (ΔE ≈ +0.4 eV) compared with the catalyst before OER, indicating an increase in the average oxidation state of Ir under extreme high current density. Nevertheless, the absorption‐edge energy of the post‐OER sample remains lower than that of the IrO_2_ reference, suggesting that the Ir species do not undergo complete oxidation to IrO_2_ and remain atomically dispersed within the CoFeCr LDH rather than forming bulk IrO_2_.

**FIGURE 4:**
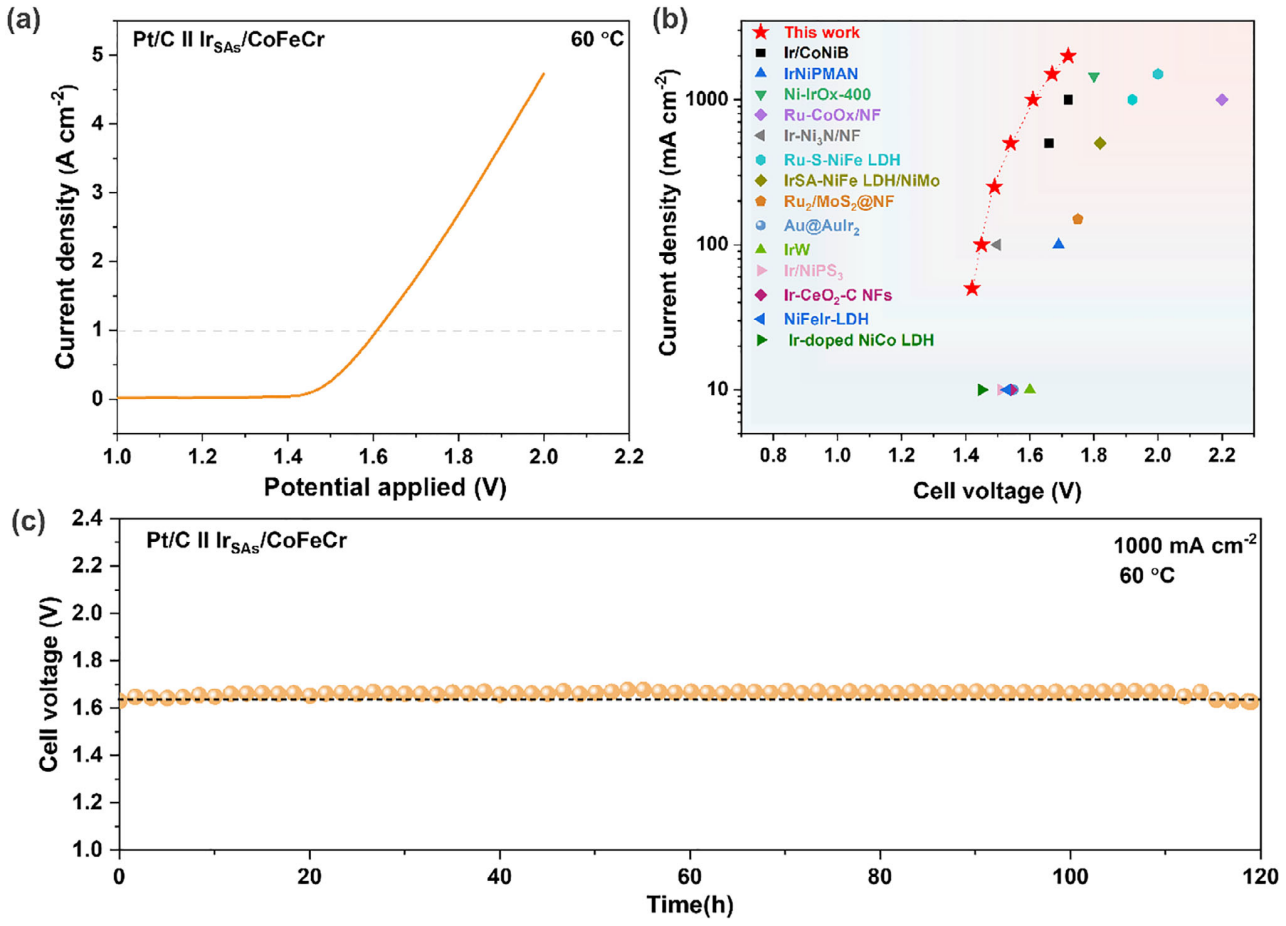
(a) LSV polarisation curves of overall water splitting performance of the assembled alkaline water electrolyser with a Pt/C‖Ir_SAs_/CoFeCr, (b) Comparison the cell voltage of Ir_SAs_/CoFeCr in AEMWEs electrolyser with other electrocatalysts [31, 32, 33, 34, 35, 36, 37, 38, 39, 40, 41, 42, 43, 44, 45, 46, 47, 48, 49, 50], (c) Long term stability test of overall water stability at 1 A cm^−2^.

### 2.4 Understanding the Role of Different Elements

To obtain the in‐depth insight into the structural evolution and electrocatalytic mechanism, determining the role of different elements and identifying the active sites are important. The schematic of in situ XAS is shown in Figure 5a. The in situ XANES at the Co K‐edge, and Ir L_3_‐edge for Ir_SAs_/CoFeCr during OER process was conducted to evaluate the real active sites and the change in valence states. XANES spectra at the 3d element K‐edge are widely recognised for their sensitivity to valence state changes. The in situ Co‐*K* XANES spectra catalyst under OER shown in Figure 5b exhibit that by increasing the applied voltage, the Co‐*K* XANES spectra of Ir_SAs_/CoFeCr gradually shifts to higher energies at 1.5 V, suggesting a transition from the lower valance of Co to higher valance. Similarly, the in situ Ir‐*L*_3_ XANES spectra also exhibit the increase in binding energy and valance state with increasing applied potential up to 1.5 V (Figure 5c). This increase in binding energy of XANES spectra for both Ir and Co by increasing the potential compared to that of open circuit potential (OCP) implies the formation of oxygen intermediate species on Co and Ir sites and the more chemisorption of OH^−^ and H_2_O [52]. Therefore, the in situ XANES of Co‐*K* and Ir‐*L_3_* XANES spectra reveal that both Co and Ir ions are OER‐active sites in Ir_SAs_/CoFeCr which are in agreements with ECSA results.

**FIGURE 5:**
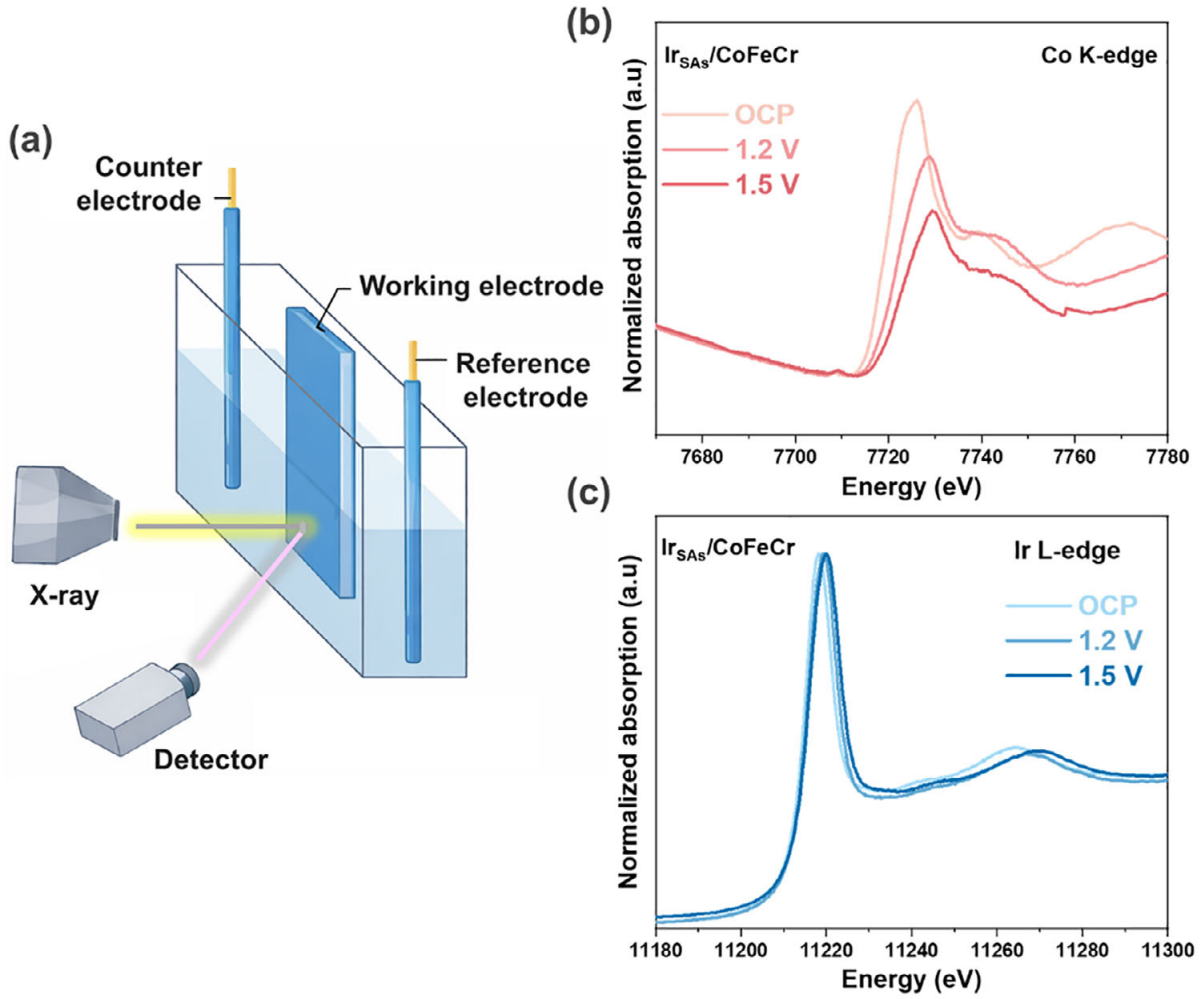
(a) Schematic of in situ XAS, and (b,c) Co K‐edge and Ir L‐edge of in situ XANES spectra of Ir_SAs_/CoFeCr at different applied voltages.

### 2.5 Unravelling the OER Mechanisms of the Electrocatalysts

The substantial reduction in the Tafel slope of Ir_SAs_/CoFeCr compared to the control catalysts (Figure 3b) suggests possible changes and modification in OER reaction kinetics and rate determining steps [53, 54]. To obtain a deeper understanding of the structural evolution and electrocatalytic mechanism during the OER process, different electrochemical experiments were conducted. The catalytic performance of Ir_SAs_/CoFeCr and Ir_SAs_/CoFe catalysts at 1.55 V under different pH values ranging from 12.5 to 14.0 was evaluated. The OER activity of Ir_SAs_/CoFe and Ir_SAs_/CoFeCr significantly enhances by increasing the pH value, implying the strong pH dependency of the catalysts (Figure S12). The correlation between OER activity and pH values was evaluated according to the proton reaction order on the RHE scale (ρ^RHE^, ρ^RHE^  =  ∂log(j)/∂pH) representing the dependence of OER reaction kinetics on proton activity [53]. The higher ρ^RHE^ indicates a stronger pH‐dependent OER activity, likely attributed to a greater degree of decoupled proton‐electron transfer during the potential‐determining step (PDS), specifically the deprotonation of ^*^OOH [10, 55, 56, 57]. The proton reaction orders (ρ^RHE^) is calculated to be 1.12 and 1.08 for Ir_SAs_/CoFeCr and Ir_SAs_/CoFe at 1.55 V (vs RHE), respectively (Figure 6a), indicating the strong pH‐dependent property, suggesting that the catalysts follow the LOM rather than the traditional AEM during OER [58, 59]. To directly confirm the LOM pathway, ^18^O isotope labeling differential electrochemical mass spectrometry (DEMS) measurements were also applied. First, Ir_SAs_/CoFeCr was electrochemically activated in a 0.1 M KOH electrolyte containing H_2_^18^O, followed by rinsing with H_2_^16^O after the labeling process. The ^18^O isotope‐labelled catalysts were then tested in a 1.0 M KOH electrolyte containing via cyclic voltammetry (CV), while the evolved gaseous products were analysed using mass spectrometry. The presence of ^34^O_2_ signals indicates direct ^16^O‐^18^O coupling, where ^16^O originates from water and ^18^O is derived from the lattice oxygen [60, 61]. The significantly stronger signal intensity of ^34^O_2_ and ^36^O_2_ than that for ^32^O_2_ (Figure 6b) represents that the oxygen products primarily originate from one ^18^O atom from the lattice oxygen and one ^16^O from the electrolyte [29, 62, 63, 64]. Based on the above analyses, the OER pathway of the Ir_SAs_/CoFeCr is confirmed to be LOM that involves the direct participation of lattice oxygen (O^2−^) in O_2_ evolution [6]. In the LOM pathway (Figure 6c), the Ir_SAs_/CoFeCr catalyst undergoes oxidation, leading to the partial removal of lattice oxygen (O^2−^) and the creation of oxygen vacancies. Hydroxide ions (OH^−^) from the electrolyte then adsorb onto these vacancy sites, forming metal‐hydroxyl intermediates (M─OH). Further oxidation facilitates the formation of a peroxo‐like intermediate (M−OOH), which plays a crucial role in O_2_ evolution. Finally, the desorption of O_2_ gas regenerates the oxygen vacancies, ensuring the continuous progression of the reaction cycle, (Figure 6c) [6]. The higher pH dependency value of Ir_SAs_/CoFeCr indicates Ir incorporation and Cr doping activates lattice oxygen, enhances proton‐electron decoupling and facilitates a greater contribution from the LOM mechanism and regeneration of lattice oxygen. To further confirm the activation of the LOM during OER, in situ Raman spectroscopy was conducted under increasing applied potentials. As shown in Figure S13, the characteristic Raman bands at approximately 400–600 cm^−1^, assigned to the M─O and M─OOH vibrations [65, 66]. More importantly, a distinct Raman band emerges at approximately 1013 cm^−1^ and continuously increases in intensity during the reaction. This band is attributed to surface superoxide (MOO^−^/O_2_^2−^) species, which is recognised as a characteristic spectroscopic of the LOM [67]. The appearance and progressive enhancement of this superoxide species indicate the formation of O─O bonded intermediates involving lattice oxygen further confirming the activation of the LOM during OER [67, 68]. Considering the LOM pathway and its distinct steps in oxygen evolution and the enhanced both activity and stability by incorporating Ir single atoms and Cr doping, DFT calculations were performed to elucidate how this mechanism enhances the OER performance of Ir_SAs_/CoFeCr.

**FIGURE 6:**
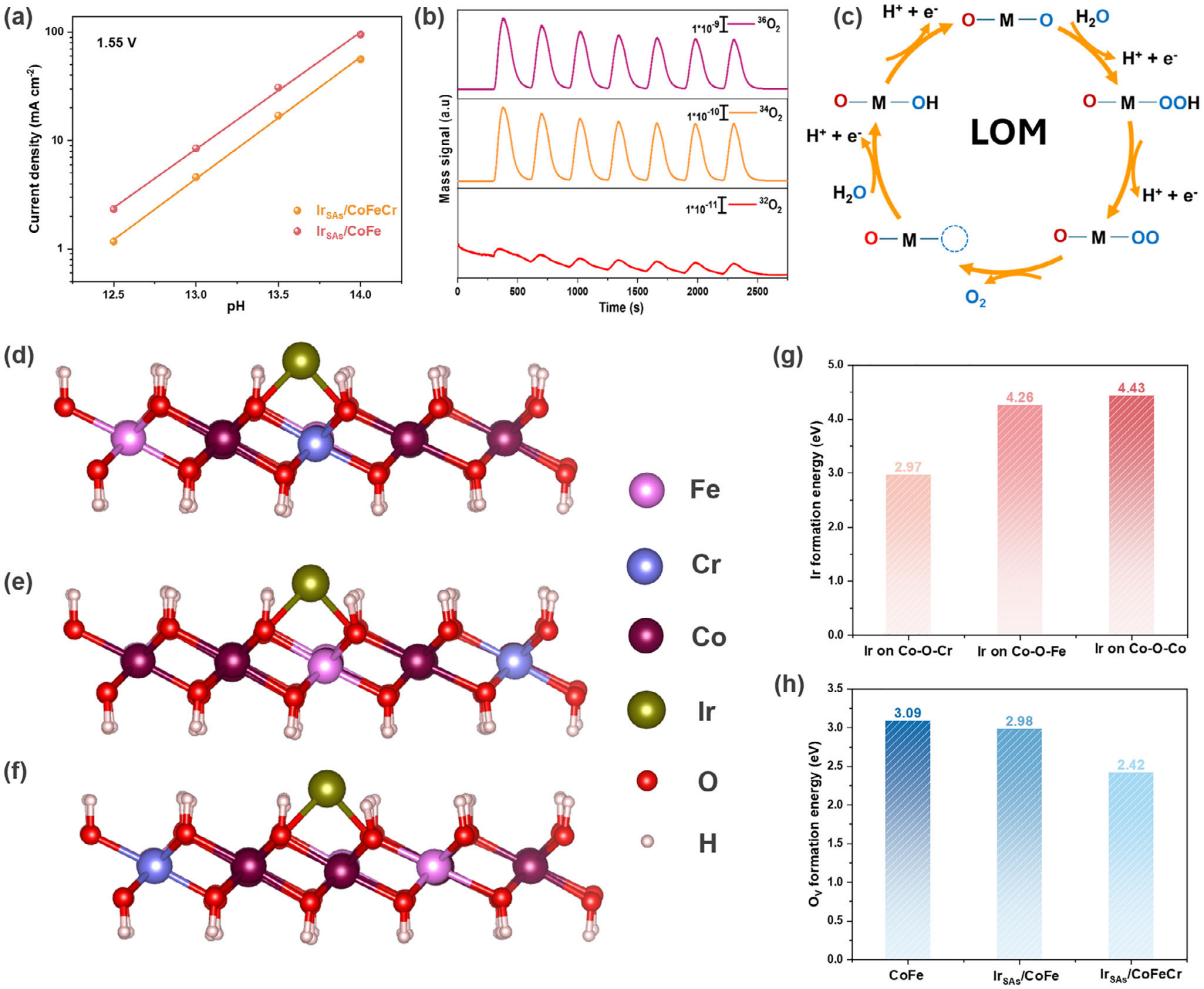
(a) pH dependency of Ir_SAs_/CoFe and Ir_SAs_/CoFeCr, (b) DEMS analysis, (c) LOM pathway, (d,e) Proposed models for Ir_SAs_/CoFeCr (d: Ir on Co─O─Cr, e: Ir on Co─O─Fe and f: Ir on Co─O─Co), (g) Ir formation energy of Ir_SAs_/CoFeCr, and (h) O_v_ formation energy of Ir_SAs_/CoFeCr, Ir_SAs_/CoFe and CoFe.

To understand how Ir is anchored on the CoFeCr, we calculated the formation energy of Ir_SAs_/CoFeCr with three possibilities of Ir is anchored on Co─O─Fe or Co─O─Cr or Co─O─Co (Figure 6d–f). Comparison the formation energy of Ir_SAs_/CoFeCr (Figure 6g) shows that the formation energy of Ir_SAs_/CoFeCr where Ir is anchored on the Co─O─Cr (2.97 eV) is much less compared to the structure in which Ir binds with O from Co─O─Fe (4.26 eV) or Co─O─Co (4.43 eV). The lower formation energy of Ir_SAs_/CoFeCr where Ir binds with O from Co─O─Cr indicates the most favourable configuration of Ir incorporation, which is used for further calculation of oxygen vacancy formation energy to compare with the control catalysts. The formation of oxygen vacancies plays a pivotal role in facilitating the LOM and the regeneration of lattice oxygen [6] while the oxygen vacancy formation energy of Ir_SAs_/CoFe and Ir_SAs_/CoFeCr is lower than that of CoFe (Figure 6h). The trend follows the order: Ir_SAs_/CoFeCr < Ir_SAs_/CoFe < CoFe. This suggests that incorporating Ir and doping Cr facilitate the formation of oxygen vacancies, thereby effectively resulting in a more efficient LOM mechanism in Ir_SAs_/CoFeCr with improved OER activity [9].

Combining the experimental results and theoretical calculations, Ir_SAs_/CoFeCr follows the LOM pathway in which Ir binds with O from Co─O─Cr. Ir and Co are determined as active sites for alkaline OER and Cr modifies the electronic structure of Ir_SAs_/CoFeCr, promoting electronic coupling between Ir and the LDH support to enhance the activity and structural stability. Besides, the results also reveal that the simultaneous incorporation of Ir and Cr doping effectively activates the participation of lattice oxygen in CoFe LDH.

## 3 Conclusion

In summary, we demonstrate that the incorporation of Ir SAs and Cr doping into CoFe LDH effectively promote a LOM pathway, in which Ir binds with O in the Co─O─Cr framework to simultaneously enhance OER activity and stability. The resulting Ir_SAs_/CoFeCr LDH exhibits exceptional OER performance, achieving an overpotential of 252 mV at 10 mA cm^−2^ and stable operation for 100 h at 500 mA cm^−2^. Combined experimental and theoretical results reveal that Ir SAs, Co centres, and lattice oxygen collectively participate in the LOM pathway, while Cr plays a critical role in modulating the electronic structure. Specifically, Cr induces electron enrichment of neighbouring Co and Fe sites and strengthens electronic coupling between Ir and the LDH support, thereby suppressing over‐oxidation and facilitating lattice oxygen regeneration during OER. The Ir_SAs_/CoFeCr LDH further demonstrates strong potential for application in AEMWEs, delivering a low cell voltage of 1.61 V at 1 A cm^−2^ and stable operation for over 120 h with a negligible decay rate of 0.04 mV/h under industrially relevant current densities. Beyond the specific system, this work highlights the importance of resolving the interplay between local coordination environment, electronic structure, and catalytic function using element‐specific and operando‐sensitive techniques. Such insights are essential for the rational design of electrocatalysts that achieve both high activity and long‐term stability, particularly for systems associated with LOM.

## 4 Experimental Section

### 4.1 Preparation of CoFeCr

A representative synthesis of CoFeCr was performed as follows: Co (NO_3_)_2_·6H_2_O (4 mmol), Fe(NO_3_)_3_·9H_2_O (1.5 mmol) and Cr (NO_3_)_3_·9H_2_O (0.5 mmol) were dissolved in 40 mL of deionised water to obtain a homogeneous solution (solution A, Co:Fe+Cr  =  2:1). Simultaneously, a separate aqueous solution containing Na_2_CO_3_ (3 mmol) and NaOH (21 mmol) in 40 mL of deionised water was prepared (Solution B). Subsequently, solutions A and B were simultaneously added dropwise into a beaker containing 80 mL of deionised water under constant stirring, until the pH of the mixture reached 9 followed by continuous stirring for 24 h. The brown precipitates were formed and collected by centrifugation, followed by three times washing with deionised water and ethanol. The product was then dried at 60 °C under atmospheric pressure overnight to obtain CoFeCr. Besides, CoFe was also synthesised using a similar procedure with Co:Fe ratio of 2:1 for comparison.

### 4.2 Preparation of Ir_SAs_/CoFeCr

Hydrogen hexachloroiridate(IV) hydrate (H_2_IrCl_6_, 0.1 mmol) was dissolved in 40 mL of deionised water containing 0.01 M NaOH. Then, 100 mg of the as‐synthesised CoFeCr powder was added to the solution, and the mixture was stirred at room temperature. The dark brown precipitate was collected by centrifugation, washed three times with deionised water and ethanol, and then dried in a vacuum oven at 60 °C overnight. Similarly, Ir_SAs_/CoFe was also obtained by following the same preparation method.

### 4.3 Characterisation

XRD was performed using a PANalytical Xpert Multipurpose XRD system with a 3 kW Co anode. High‐angle annular dark‐field scanning transmission electron microscopy (HAADF‐STEM) was performed using an aberration‐corrected JEOL JEM‐ARM300F2 STEM system operated at an accelerating voltage of 300 kV. HAADF imaging was conducted with a camera length of 8 cm, corresponding to a minimum annular dark‐field (ADF) collection angle of approximately 75 mrad. Elemental analysis via energy‐dispersive x‐ray spectroscopy (EDS) was conducted using two JEOL EDX silicon drift detectors (SDDs), offering a combined detection area of 158 mm^2^ dual JEOL EDX SDD detectors with a total collection area of 1.4 sr. The metal loading of Ir and Cr contents were determined using inductively coupled plasma mass spectrometry (ICP‐MS) and the amount of Co and Fe analysed by inductively coupled plasma optical emission spectroscopy (ICP‐OES). X‐ray photoelectron spectroscopy (XPS) measurements were performed using a Thermo ESCALAB 250Xi spectrometer equipped with Al Kα radiation. X‐ray absorption spectroscopy (XAS) samples were prepared according to the guidelines provided by the Australian Synchrotron XAS Beamline. Specifically, the powdered catalysts were mixed and grinded with cellulose with a mass ratio suitable for fluorescence measurements. Ir L‐edge and Co, Fe and Cr K‐edge XAS were measured at the Australian Synchrotron XAS Beamline and the Medium Energy XAS (MEX‐1) Beamline respectively. The electron beam energy was maintained at 3.0 GeV with a current of 200 mA. Data were collected in fluorescence mode using an 18‐element solid‐state Ge detector. Energy calibration was carried out using the first inflection point of Ir foil for the Ir L‐edge, Co foil for the Cr K‐edge, Fe foil for the Cr K‐edge and Cr foil for the Cr K‐edge. XAS data were processed using Pyspline for background subtraction and Artemis for fitting the extended x‐ray absorption fine structure (EXAFS) region.

### 4.4 Electrochemical Measurements

Preparation of the working electrode: 5.0 mg of the electrocatalyst was dispersed in a mixture containing 0.50 mL of deionised water, 0.50 mL of ethanol, and 25 µL of 5 wt.% Nafion solution. The resulting suspension was sonicated for 30 min to obtain a homogeneous catalyst ink. Subsequently, 3.6 µL of the ink was drop‐cast onto the surface of a glassy carbon electrode, yielding a catalyst loading of 0.238 mg·cm^−2^. Electrochemical measurements were carried out in a three‐electrode setup, using a platinum wire as the counter electrode and a Hg/HgO as the reference electrode. Linear sweep voltammetry (LSV) was performed in 1 M KOH with a scan rate of 5 mV·s^−1^. To ensure stability, at least ten LSV scans were conducted before collecting the final data. All potentials were converted to the reversible hydrogen electrode (RHE) scale using the following equation:

$$E_{\mathrm{RHE}}=E_{\mathrm{Hg}/\mathrm{HgO}}+0.059\times \mathrm{pH}+0.098\;V$$

Tafel slopes were calculated by linear fitting of the plot of overpotential vs. the logarithm of current density, according to the Tafel equation:

η=a+blogj

where a corresponds to the intercept, b is the Tafel slope, η and j represent the overpotential, and the current density, respectively.

The mass activity of the catalysts was determined using the following equation:

$$mass\;activity=\frac{j}{m_{metal}}$$

where j represents the measured current density (A·cm^−2^), and m is the catalyst loading based on the metal (mg cm^−2^).

The long‐term stability of the electrocatalysts was evaluated by chronopotentiometry (CP) measurements conducted using a Metrohm Autolab electrochemical workstation equipped with an Autolab M204 potentiostat and a Booster 10 A module. The tests were performed in a three‐electrode configuration using a 2 × 2 cm^2^ piece of carbon paper, with the catalyst sprayed over a 1 × 1 cm^2^ area as a working electrode. CP measurements were carried out at a constant current density of 500 mA cm^−2^, while continuously recording the electrode potential as a function of time.

The performance decay rate was determined from the slope of the voltage‐time (*V*–*t*) curves obtained during CP tests and calculated according to:

$$\mathrm{Decay}\;\mathrm{rate}\;\left(\mathrm{mV}\;h^{-1}\right)=\frac{V_{\mathrm{end}}-V_{\mathrm{start}}}{\Delta t}$$

where *V*_start_and *V*_end_ are the initial and final cell voltages, respectively, and Δ*t* is the duration of the stability test. This parameter reflects the kinetics of performance degradation during continuous operation.

In addition, the decay ratio was used to evaluate the overall extent of performance degradation and was calculated as:

$$\mathrm{Decay}\;\mathrm{ratio}\;\left(\%\right)=\frac{V_{\mathrm{end}}-V_{\mathrm{start}}}{V_{\mathrm{start}}}\times 100\%$$

Isotope‐labeled differential electrochemical mass spectrometry (DEMS) measurements were carried out using Linglu QAS100 differential electrochemical mass spectrometry coupled to an Autolab potentiostat (PGSTAT204, Metrohm). H_2_^18^O, 97 atom% ^18^O) was purchased from Innochem. A conventional three‐electrode configuration was employed, consisting of a saturated Ag/AgCl reference electrode, a Pt wire counter electrode, and a glassy carbon working electrode onto which the catalyst ink was drop‐cast. To introduce ^18^O isotopes, cyclic voltammetry (CV) was first performed for five cycles between 1.1 and 1.9 V at a scan rate of 5 mV s^−^^1^ in ^18^O ‐enriched 1.0 M KOH electrolyte. After the labelling step, the electrode surface was thoroughly rinsed with H_2_^16^O, and the same CV protocol was subsequently conducted in ^16^O ‐containing 1.0 M KOH electrolyte. During the measurements, the ion currents corresponding to gaseous products with different mass‐to‐charge ratios were continuously monitored in real time, enabling direct evaluation of lattice‐oxygen participation from the catalyst during the OER. In situ Raman was conducted with Renishaw inVia Raman microscope (Qontor) with the laser wavelength of 514 nm.

Anion exchange membrane water electrolyzer (AEMWE) tests were conducted using Ir_SAs_/CoFeCr as anode and Pt/C as the cathode. The anode catalyst ink was sprayed onto stainless steel fiber paper gas diffusion layers (GDL, 1 × 1 cm^2^, Fuel Cell Store), while the cathode catalyst was deposited onto nickel fiber paper GDLs (1 × 1 cm^2^, Fuel Cell Store). The anode and cathode catalyst loadings were controlled to be 1 mg cm^−2^ and 0.5 mg cm^−2^, respectively. The GDL‐loaded electrocatalysts were fixed with Nafion 212 membrane. The anode and cathode were circulated with 1 M KOH (flow rate: 40 mL min^−1^) and the cell temperature was maintained at 60 °C during the test.

### 4.5 DFT Method

Density functional theory (DFT) calculations were performed using the Projector Augmented Wave (PAW) method as implemented in the Vienna Ab initio Simulation Package (VASP) [69, 70, 71, 72]. The calculations were completed with a plane‐wave cut‐off energy of 500 eV and Gamma k‐points mesh of 3 × 3 × 1. The electronic self‐consistent calculation was converged to 1 × 10^−5^ eV and ionic relaxation steps were performed using the conjugate‐gradient method (IBRION = 2) and continued until the total force on each atom dropped below a tolerance of 2 × 10^−2^ eV/Å. The generalised gradient approximation (GGA) was used for the exchange correlation functionals as parameterised by Perdew‐Burke‐Ernzerhof (PBE) [73]. The dispersion correction was also included in this study by using DFT D‐3 method [74].

## Author Contributions

P.E. conceived experiments, conducted data analysis, and wrote the first manuscript. S.Z., X.L, and R.A supervised the project and revised the manuscript. J.Y. helped with DFT calculations and analysis. R.W helped with TEM analysis. Y.Zh, M.Zh, TS Bui and B.J helped with XAS measurements and analysis. H.X. assisted with AEMWEs experiments. Zh.T and J.B assisted with DEMS test. J.C assisted with data analysis and visualisation. All authors contributed to the manuscript revision. All authors have given approval to the final version of the manuscript.

## Conflicts of Interest

The authors declare no conflict of interest.

## Supporting information




**Supporting File**: advs77198‐sup‐0001‐SuppMat.docx.

## Data Availability

The data that support the findings of this study are available from the corresponding author upon reasonable request.
