# Supplementary material for: Stabilising the Lattice Oxygen Oxidation Mechanism Through Iridium Single Atoms in Chromium‐Doped Cobalt Iron Layered Double Hydroxides
Source: Adv Sci (Weinh). 2026 Aug 14;13(61):e77198. doi: 10.1002/advs.77198 (PMC13474180; doi:10.1002/advs.77198)
Supplement: Supplementary file 1 [file ADVS-13-e77198-s001.docx]

**Stabilising the Lattice Oxygen Oxidation Mechanism through Iridium Single Atoms in Chromium-Doped Cobalt Iron Layered Double Hydroxides**

Parisa Eskandari^a^, Shujie Zhou^a^*, Jodie Yuwono^b^, Yufei Zhao^c^, Hanyu Xu^a^, Zhihong Tian^d^, Jingwen Ba^d^, Ming Zhang^a^, Bernt Johannessen^e^, Thanh Son Bui ^a^, [Richard F. Webster](https://advanced.onlinelibrary.wiley.com/authored-by/Webster/Richard+F.)^f,g^, Jing Chu^a^, Xunyu Lu^a^, Rose Amal^a^*

^a^ School of Chemical Engineering, University of New South Wales, UNSW Sydney, Kensington, NSW 2052, Australia

^b^ School of Chemical Engineering, Adelaide University, Adelaide, SA 5005, Australia

^c^ Centre for Clean Energy Technology, University of Technology Sydney, Broadway, NSW 2007, Australia

^d^ Engineering Research Center for Nanomaterials, Henan University, Kaifeng 475004, P. R. China

^e^ Australian Synchrotron-ANSTO, Clayton, VIC 3168, Australia

^f^ Electron Microscope Unit, Mark Wainwright Analytical Centre, UNSW Sydney, Kensington, NSW 2052, Australia

^g^ School of Materials Science and Engineering, UNSW Sydney, Kensington, NSW 2052, Australia

Email addresses: [shujie.zhou@unsw.edu.au](mailto:shujie.zhou@unsw.edu.au) (S. Zhou), [r.amal@unsw.edu.au](mailto:r.amal@unsw.edu.au) (R.Amal)

**Supporting figures:**

**Fig. S1**. Schematic of the synthesis method of CoFeCr and Ir_SAs_/CoFeCr LDH.

**Fig. S2** XRD patterns of CoFe LDH, CoFeCr LDH, Ir_SAs_/CoFe LDH and Ir_SAs_/CoFeCr LDH

**Fig.S3** HRTEM image of different regions of Ir_SAs_/CoFeCr for the lattice spacing of LDH

**Fig. S4** a,c) Ir L-edge EXAFS (point) and the curve fit (line) for Ir_SAs_/CoFe and Ir_SAs_/CoFeCr and b, d) the curve fit (line) for Ir_SAs_/CoFe and Ir_SAs_/CoFeCr, shown in R-space (FT magnitude and imaginary component). The data are k3-weighted and not phase-corrected.

**Fig. S5.** a) XANES and fitting results of Ir_SAs_/CoFeCr and b)Ir_SAs_/CoFe.

**Fig. S6**. a, and b) XPS spectra of Co and Fe for CoFe, CoFeCr, and Ir_SAs_/CoFeCr

**Fig.S7**. EIS of CoFe, CoFeCr, Ir_SAs_/CoFe and Ir_SAs_/CoFeCr

**Fig. S8**. The corresponded CV curves for a) CoFe, b) CoFeCr, c) Ir_SAs_/CoFe and d) Ir_SAs_/CoFeCr, and e) Electrochemical double-layer capacitance (C_dl_)

**Fig. S9.** EIS of Pt/C‖Ir_SAs_/CoFeCr

**Fig. S10**. Stability test of AEMWE using Pt/C|| Ir_SAs_/CoFeCr at 2 A.cm^-2^

**Fig. S11.** XANES spectra of Ir_SAs_/CoFeCr at Ir L-edge before and after AEMWE reaction.

**Fig. S12.** LSV of different pH for Ir_SAs_/CoFeCr and Ir_SAs_/CoFe

**Fig. S13.** In-situ Raman for Ir_SAs_/CoFeCr at 1.5V vs. RHE

**Table S1.** Comparison the cell voltage of Ir_SAs_/CoFeCr in AEM electrolyser with other electrocatalysts.

| Electrocatalyst | Current density (mA cm^-2^) | Cell voltage (V) | Ref |
| --- | --- | --- | --- |
| Ir_SAs_/CoFeCr | 100 | 1.45 | This work |
| Ir_SAs_/CoFeCr | 500 | 1.54 | This work |
| Ir_SAs_/CoFeCr | 1000 | 1.6 | This work |
| Ir@Ni-NDC | 10 | 1.46 | ^[1]^ |
| Au@AuIr_2_ | 10 | 1.55 | ^[2]^ |
| Ir@N-G-600 | 10 | 1.566 | ^[3]^ |
| IrW | 10 | 1.6 | ^[4]^ |
| FF–NaCl–Ir–P | 10 | 1.47 | ^[5]^ |
| Ir/NiPS_3_ | 10 | 1.51 | ^[6]^ |
| ZnIr(OH)_6_ | 10 | 1.476 | ^[7]^ |
| Ir-NR/C | 10 | 1.57 | ^[8]^ |
| Ir-CeO_2_-C NFs | 10 | 1.54 | ^[9]^ |
| Ir_1_Ni@MoO_2_ SAAs | 10 | 1.52 | ^[10]^ |
| NiFeIr-LDH | 10 | 1.53 | ^[11]^ |
| Ir-doped NiCo LDH | 10 | 1.45 | ^[12]^ |
| IrNi PMAN | 100 | 1.69 | ^[13]^ |
| Ir-Ni_3_N/NF | 100 | 1.498 | ^[14]^ |
| Ru_2_/MoS_2_@NF | 150 | 1.75 | ^[15]^ |
| Ir_SA_-NiFe LDH/NiMo | 500 | 1.82 | ^[16]^ |
| Ir/CoNiB | 500 | 1.66 | ^[17]^ |
| Ir/CoNiB | 1000 | 1.72 | ^[17]^ |
| Ni-IrO_x_-400 | 1454.8 | 1.8 | ^[18]^ |
| Ru-CoO_x_/NF | 1000 | 2.2 | ^[19]^ |
| Ru-S-NiFe LDH | 1000 | 1.92 | ^[20]^ |
| Ru-S-NiFe LDH | 1500 | 2 | ^[20]^ |

**Table S2.** Modelled parameters of the Nyquist plots in Fig.S8

| Catalyst | R_1_(Ω) | R_2_(Ω) | R_3_(Ω) |
| --- | --- | --- | --- |
| Pt/C‖Ir_SAs_/CoFeCr | 0.074 | 0.021 | 0.025 |

Table S3. ICP-MS analysis of dissolved metal composition after AEMWE.

| Element | Dissolved amount (mg) | Dissolved (%) |
| --- | --- | --- |
| Co | 0.000434 | 0.017 |
| Ir | 0.02590 | 1.04 |
| Cr | 0.10284 | 4.11 |

**References**

[1]	J. Yang, Y. Shen, Y. Sun, J. Xian, Y. Long, G. Li, *Angewandte Chemie* **2023**, 135, e202302220.

[2]	H. Wang, Z.-n. Chen, D. Wu, M. Cao, F. Sun, H. Zhang, H. You, W. Zhuang, R. Cao, *Journal of the American Chemical Society* **2021**, 143, 4639.

[3]	L. Yi, B. Feng, N. Chen, W. Li, J. Li, C. Fang, Y. Yao, W. Hu, *Chemical Engineering Journal* **2021**, 415, 129034.

[4]	L. Fu, X. Hu, Y. Li, G. Cheng, W. Luo, *Nanoscale* **2019**, 11, 8898.

[5]	Z. Zhao, W. Jin, L. Xu, C. Wang, Y. Zhang, Z. Wu, *Journal of Materials Chemistry A* **2021**, 9, 12074.

[6]	Y. Liu, L. Li, L. Wang, N. Li, X. Zhao, Y. Chen, T. Sakthivel, Z. Dai, *Nature communications* **2024**, 15, 2851.

[7]	S. Liu, Y. Zhang, X. Mao, L. Li, Y. Zhang, L. Li, Y. Pan, X. Li, L. Wang, Q. Shao, *Energy & Environmental Science* **2022**, 15, 1672.

[8]	F. Luo, L. Guo, Y. Xie, J. Xu, K. Qu, Z. Yang, *Applied Catalysis B: Environmental* **2020**, 279, 119394.

[9]	X. Chen, W. Liao, M. Zhong, J. Chen, S. Yan, W. Li, C. Wang, W. Chen, X. Lu, *Nano Research* **2023**, 16, 7724.

[10]	B. Wang, J. Li, D. Li, J. Xu, S. Liu, Q. Jiang, Y. Zhang, Z. Duan, F. Zhang, *Advanced materials* **2024**, 36, 2305437.

[11]	S. Jung, R. A. Senthil, A. Min, A. Kumar, C. J. Moon, G. H. Jeong, T. W. Kim, M. Y. Choi, *Journal of Materials Chemistry A* **2024**, 12, 8694.

[12]	R. Fan, Q. Mu, Z. Wei, Y. Peng, M. Shen, *Journal of Materials Chemistry A* **2020**, 8, 9871.

[13]	Z.-H. Zhou, W.-H. Li, Z. Zhang, X.-C. Zhao, W. Cao, Q.-S. Huang, *International Journal of Hydrogen Energy* **2023**, 48, 8440.

[14]	Y. Chen, J. Meng, M. Xu, L. Qiao, D. Liu, Y. Kong, X. Hu, Q. Liu, M. Chen, S. Lyu, *Advanced Functional Materials* **2025**, 35, 2413474.

[15]	P. Li, S. Luo, Z. Xiong, H. Xiao, X. Wang, K. Peng, X. Xie, Z. Zhang, G. Deng, M. Yang, *Molecular Catalysis* **2025**, 570, 114710.

[16]	Y. Wu, M. Chen, H. Sun, T. Zhou, X. Chen, G. Na, G. Qiu, D. Li, N. Yang, H. Zheng, *Applied Catalysis B: Environment and Energy* **2025**, 360, 124548.

[17]	C. Wang, P. Zhai, M. Xia, Y. Wu, B. Zhang, Z. Li, L. Ran, J. Gao, X. Zhang, Z. Fan, *Angewandte Chemie International Edition* **2021**, 60, 27126.

[18]	D. H. Park, M. H. Kim, H. J. Lee, W. J. Lee, J. H. Byeon, J. H. Kim, J. S. Jang, K. W. Park, *Advanced Materials Interfaces* **2022**, 9, 2102063.

[19]	D. Wu, D. Chen, J. Zhu, S. Mu, *Small* **2021**, 17, 2102777.

[20]	Y. Zhu, J. Wang, G. Weiser, M. Klingenhof, T. Koketsu, S. Liu, Y. Pi, G. Henkelman, X. Shi, J. Li, *Advanced Energy Materials* **2025**, 2500554.
